# Early exposure-response modeling of an interferon-beta monoclonal antibody (dazukibart) in adults with dermatomyositis

**DOI:** 10.1007/s10928-026-10022-1

**Published:** 2026-03-24

**Authors:** John P. Prybylski, Jing ‘Daisy’ Zhu, Christopher Banfield, Arnab Mukherjee, Vivek Purohit

**Affiliations:** 1 Pharmacometrics and Systems Pharmacology, Pfizer, Groton, CT USA; 2 Present Address: Clinical Pharmacology and Pharmacometrics, Biogen, Cambridge, MA USA; 3 Clinical Pharmacology, Pfizer, Collegeville, PA USA; 4 Clinical Pharmacology, Pfizer, Cambridge, MA USA; 5 Clinical Pharmacology, Pfizer, Groton, CT USA; 6 Present Address: PK Sciences, Novartis, Cambridge, MA USA; 7 Present Address: CB Pharma Consulting Services LLC, Cambridge, MA USA; 8 Present Address: Clinical Pharmacology, Incyte, Wilmington, DE USA

**Keywords:** Dermatomyositis, Dazukibart, Exposure-response, CDASI, TIS

## Abstract

Dermatomyositis (DM) is a rare and debilitating inflammatory disease associated with muscle weakness and skin manifestations. As with all rare diseases, clinical trials in DM are challenging due to the small number of patients available for study, and DM patients may even present with skin- or muscle-predominant disease. A Phase 2 study of an anti-interferon-beta monoclonal antibody (dazukibart) in DM was recently completed. Most trial data were collected in skin-predominant DM patients, measuring the Cutaneous Dermatomyositis Disease Area and Severity Index (CDASI); the Total Improvement Score (TIS) was only measured in a small subset of muscle-predominant DM patients. Because TIS is a more holistic endpoint, planning for further dazukibart development depended on a robust understanding of TIS response. This analysis aimed to develop an exposure-response model for dazukibart in DM patients. The model was intended to describe the timecourses of all relevant clinical responses, including CDASI and TIS, using the available data to collectively inform the exposure-response relationships. The model provided evidence for a TIS response in DM patients treated with dazukibart that was consistent with the observed data and supportive of further development. The model used here could be applied directly to model-based meta-analysis of other DM trials, and the general approach can be used in rare diseases with multiple endpoints.

**Trial registration numbers: **NCT03181893, NCT02766621.

**Supplementary Information:**

The online version contains supplementary material available at 10.1007/s10928-026-10022-1.

## Introduction

Dermatomyositis (DM) is a rare and debilitating inflammatory disease, causing muscle weaknesses associated with other types of idiopathic immune myopathies (IIM), in addition to extramuscular manifestations in the skin [1, 2]. It can emerge at any age and appears to affect more women than men [3]. Typical treatments include corticosteroids and while other inflammation and immunology drugs are used in the disease state, intravenous immune globulin (IVIG) is the only non-steroidal treatment approved for this indication by the United States Food and Drug Administration (several alternatives are approved by other regulatory agencies). The approval of IVIG was largely based on successful completion of ProDERM [4].

Dazukibart is a monoclonal antibody (mAb) for interferon-beta (IFN_β_). IFN_β_ is a key cytokine in the pathology of DM, which is evidenced by the correlation of IFN_β_levels or IFN_β_ gene signature (quantifying production of IFN_β_- responsive mRNA) and disease activity [5]. This recognized role has led to investigations targeting the Janus kinase pathway involved in type-I interferon signaling, or mAbs targeting other type-I interferons [6–8]; these treatments have had mixed success, limited in part by the recruitment difficulties for a rare disease, but also potentially by the lack of pharmacological specificity.

DM manifests with both skin and muscle pathology, but amyopathic DM accounts for around 20% of all cases [9]. Since both classical DM and amyopathic DM would present with skin dysfunction, disease scoring for monitoring and treatment has often focused on Cutaneous Dermatomyositis Disease Area and Severity Index (CDASI) [10]. However, this score does not track muscle outcomes, which are clinically relevant as they could lead to the lung complications of much concern in this population [11]. Thus, Total Improvement Score (TIS) is preferred to monitor holistic improvement over time [1].

The study C0251002 was initially designed to identify the cutaneous response to dazukibart in DM patients. After various stages focused on skin endpoints, eventually TIS response was measured in a small, final set of study arms. The outcomes data from this study are extensive and provide valuable insight into the longitudinal cutaneous and functional responses of DM patients. However, there are only minimal data on the TIS response, which was necessary to understand for continued development.

The present analysis aimed to develop an exposure-response (ER) model for dazukibart in DM patients, with a focus on the TIS response. The model was intended to describe the timecourses of all relevant clinical responses, using the available data to collectively inform the ER relationships. The model was then used to predict TIS response under various conditions to provide estimands of treatment effect.

## Methods

### Study design

C0251002 was a randomized, double-blind, placebo-controlled Phase 2 study in adults with DM. The design of the study was adaptive, being run in stages with interim analyses to determine the updated design for the next stage. In Stage 1, patients with skin-predominant DM (SPDM) were randomized to either 600 mg dazukibart or placebo intravenously every 4 weeks for 3 doses. In Stage 2, a lower dose of 150 mg (at the same frequency) was added, but otherwise the same design as in Stage 1 was maintained. In Amended Stage 2 (2 A), a crossover was implemented where patients were randomized into sequences switching from placebo to active or active to placebo at 12 weeks; the 150 mg and 600 mg dosing levels from Stage 2 were maintained. In Stage 3, patients with muscle-predominant DM (MPDM) were randomized to either 600 mg dazukibart or placebo every 4 weeks for 3 doses, with a crossover at 12 weeks. Efficacy in Stages 1, 2 and 2 A was assessed by the CDASI and in Stage 3 by the Total Improvement Score (TIS). TIS was not measured in Stages 1, 2, or 2 A.

### Outcomes assessments

The clinical scores considered in the analysis are listed below, with ranges and directions of improvement. Background and descriptions for these endpoints have been published previously [1, 10].

- CDASI (the Activity and Damage subscores, CDASI-A and CDASI-D): CDASI-A 0–100, CDASI-D 0–32, lower is improvement;
- 36-Item Short Form Survey (SF-36) (Mental Component and Physical Function Domain Scores, SF-36 MCS and SF-36 PFD): Both subscores 0–100, higher is improvement;
- Physician and Patient Global Assessments, PhGA and PtGA: Both 0–100, lower is improvement;
- TIS, including.
  - PtGA and PhGA, where PhGA is DM-specific: PtGA same as above, PhGA 0–10, lower is improvement;
  - 8-Group Manual Muscle Testing (MMT-8): 0–150, higher is improvement;
  - Health Assessment Questionnaire (HAQ): 0–3 (expressed as percent), lower is improvement;
  - Most abnormal muscle enzyme: Based on 90% range from natural history data of the most abnormal enzyme [1], lower is improvement;
  - Extramuscular Global Assessment (ExGA) from Myositis Disease Activity Assessment Tool (MDAAT): 0–10 (expressed as percent), lower is improvement.

The TIS is derived from the “absolute percent improvement” (change from baseline relative to the total range) of each subscore, categorized at various improvement thresholds [1]. TIS is further categorized into minimal, moderate and major improvement depending on the level; these categories are not independent, e.g. subjects with major improvement are also counted as having moderate and minimal improvement.

### Pharmacokinetic/pharmacodynamic modeling

The pharmacokinetic/pharmacodynamic (PK/PD) model was used in part to explain the relationship between dazukibart exposure and IFN_β_ serum concentrations, which was implemented as the driving mechanism for the clinical response. The PK model was typical of monoclonal antibodies, having two compartments and first order elimination [12]; there was no evidence of target-mediated drug disposition. The PD model described IFN_β_ with a quasi-steady state approximation [13]. The PK/PD model is not the primary focus of this report, but since it has not been described previously, additional details and key parameter results can be found in the Supplementary Materials.

The individual PK/PD parameters (IPP) from the PK/PD model were used in fitting the exposure-response model, making this a sequential fit of a much larger joint model in which the PK/PD and exposure-response model are combined. The limitations of the IPP approach are well-known [14] and are most relevant when a simultaneous model fit would inform some of the parameters treated as fixed to their posthoc values. It was assumed that the exposure-response portion of the model would not greatly improve prediction of bound IFN_β_, nor significantly change the conclusions from the less complex IPP approach. However, it would be worth exploring in a simpler case.

### Exposure-response modeling

The model assumed a sigmoidal relationship between change in clinical endpoints and IFN_β_ levels. The relationship had the fraction of maximum effect correlate with free IFN_β_ (P_unbound_), or target unbound by dazukibart. A shown in Eqs. 1–3, this approach effectively described a E_max_ model, where the dazukibart concentration (C) at EC_50_ is equal to the IFN_β_ quasi-steady state binding constant (K_SS_). To account for effects not explained by IFN_β_, normalizing maximum effect (E_max, norm_) was also used. The underlying assumption for the model applied to all clinical endpoints, parameterized with a shared effect (E_shared_).

1 $$\:IFN_{total}=IFN_{unbound}+IFN_{bound}$$

2 $$\:P_{unbound}=\frac{IFN_{unbound}}{IFN_{total}}=1-\frac{IFN_{bound}}{IFN_{total}}=1-\frac C{C+K_{SS}}$$

3 $$\:{E}_{shared}={E}_{max,norm}\cdot\:{P}_{unbound}$$

**Fig. 1:**
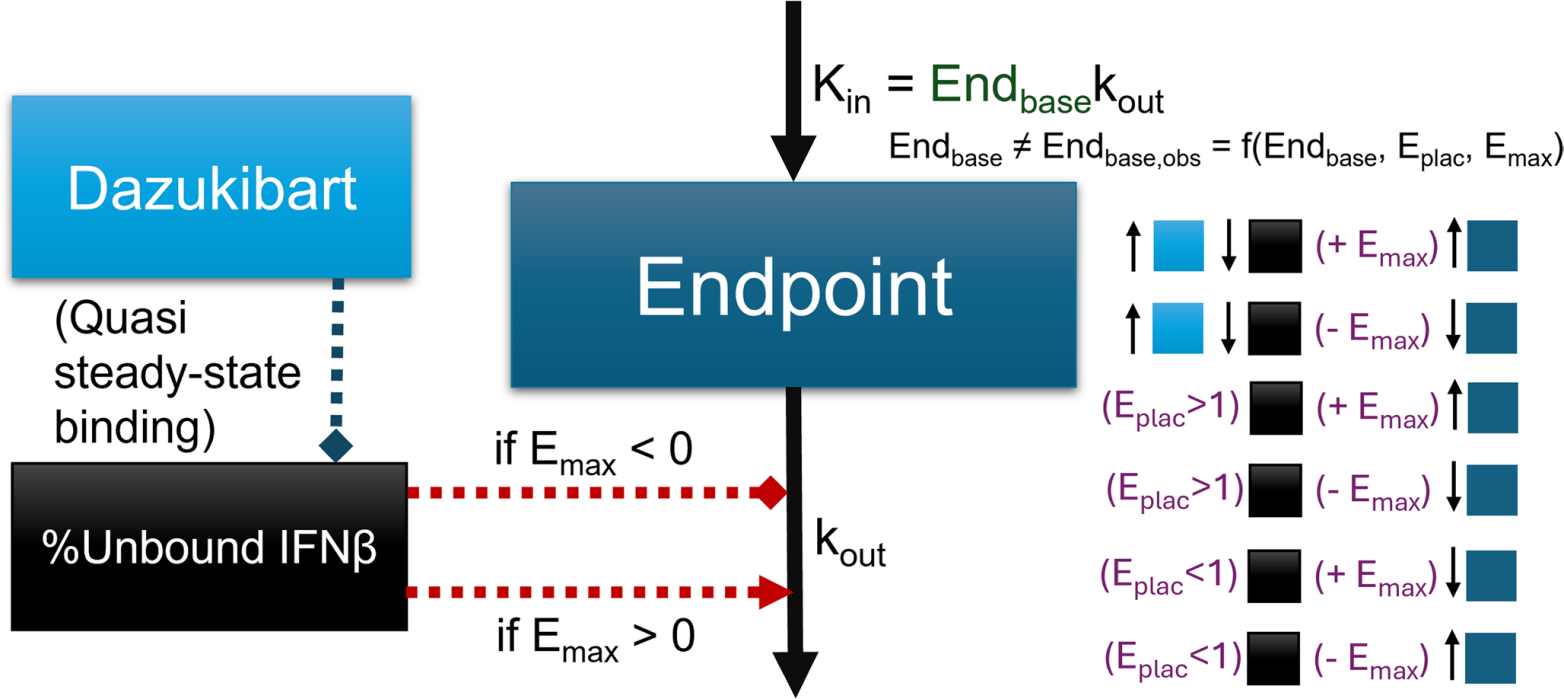
Exposure-response model diagram. The modeled exposure-response for any clinical endpoint in the model. In the diagram, states of the endpoint, dazukibart concentration and unbound fraction of IFN_β_are represented as boxes, with mass transfer for the indirect response model represented with solid arrows, and inhibition (diamond capped) and stimulation (arrow capped) represented with dashed arrows. The impact of dazukibart on IFN_β_fraction bound (or placebo effect, E_plac_) and downstream effect on endpoint increase or decrease, depending on the direction of endpoint-specific maximum effect, is also shown with abstract boxes color-coded to the matching compartments. It is emphasized that the endpoint baseline (End_base_) is different from the observed baseline (beyond model error), because the observed baseline is modeled to represent the steady-state after sustained completely unbound IFN_β_, and the direction of the modeled placebo effect. Thus, the End_base_ relevant to K_in_ can be taken as the endpoint that might be achieved with complete IFN_β_suppression and no placebo response

A diagram is available for the exposure response model in Fig. 1. For each clinical endpoint, baseline (End_base, DM, m_)^1^ and E_max,m_ were fit. The value of each E_max_ was constrained between − 1 and 1, so the direction of effect was also determined by model optimization. Placebo was modeled as a non-steady state effect where steady-state is deviated from by some factor (E_plac_) of the predicted baseline; placebo effect (E_plac_) is transformed based on the direction of effect on each endpoint (based on sign of E_max,m_) so that it is consistently modeling along or against the direction of drug effect (depending on if E_plac_ is greater than or less than 1) across all endpoints. When E_max, m_ was 0, there was no placebo effect (E_plac_^0^=1). To have the response modeled as being driven by P_unbound_, which is maximal at baseline, it was necessary to correct the baseline used for calculating kinetic parameters to the existing impact of maximal P_unbound_. The baseline correcting for IFN_β_and E_plac_ (End_base,m_) was calculated as in Eqs. (4–6) for endpoint m. The corrected baseline End_base,m_ was used to compute the endpoint input rate, k_in,m_ with shared clinical score “turnover” rate (k_out_). The endpoint models could be described as Type II/IV indirect response models (IRMs), since they were driven by inhibition and stimulation on k_out_ [15]. Since the observable baseline (End_base,DM,m_) was used as the initial endpoint condition and the IFN_β_-corrected baseline was used to calculate k_in,m_, the model can be described as being in an altered steady-state, where return to the “true” steady-state (End_base,m_) requires saturated IFN_β_binding (P_unbound_ $\:\approx\:$ 0) and a correction to the placebo-based changes to steady-state. In other words, the baseline for a DM patient is modeled as the steady-state after sustained, constant maximal effect of IFN_β_,and it would take complete saturation of IFN_β_to return to their equilibrium-driven, “healthy” baseline. In the absence of change in IFN_β_binding,E_plac_ also disturbs the baseline steady-state, but the direction depends not only on the sign of E_max,m_ but also whether E_plac_ is less than or greater than 1 (as listed in Fig. 1). This parameterization also ensures directional consistency between fitted E_max_ values and observed changes over time, while maintaining the same underlying semi-mechanistic description.

4 $$End_{base,\;m}=End_{base,\;DM,\;m}\cdot\left(1+E_{max,m}\cdot E_{max,\;norm}\right)\cdot E_{plac}^{sign(E_{max,\;m})}$$

5 $$k_{in,\;m}=k_{out}\cdot End_{base,\;m}$$

6 $$\frac{dEnd_m}{dt}=k_{in,\;m}-k_{out}\cdot End_m\cdot(1+E_{max,\;m}\cdot E_{shared})$$

Interindividual variability (IIV) was modeled as modified log-normal for endpoint baselines (where the central estimate is shifted by 1 to allow individual estimates of 0), log-normal for E_plac_, and logit-normal for E_max, norm_. Correlation of observed baselines was fitted as covariance, using a large “block” omega matrix. Because each additional endpoint thus causes at least a half-squared increase in number of fitted parameters, those with very high correlations between empirical Bayes estimates (EBEs) or other issues with identifiability were combined with fixed effects, shared, or dropped. When IIV estimates were shared, there were fixed effect parameters ($\theta_{Xc,d}$, for part of $\omega_c$ from $\omega_d$) used to compute $\eta$ as in Eq. (7), where $\eta_{1i}^\ast$ is an individual random effect estimate for subject i that is calculated from other $\eta$ terms. In cases where no independent IIV could be fitted, $\theta_{X1,\;1}$ could be considered 0, otherwise it was 1.

7 $$\:{\eta\:}_{1i}^{\mathrm{*}}={\theta\:}_{\mathrm{X}1,1}\cdot\:{\eta\:}_{1i}+{\theta\:}_{\mathrm{X}1,2}\cdot\:{\eta\:}_{2i}+{\theta\:}_{\mathrm{X}1,3}\cdot\:{\eta\:}_{3i}+\dots\:$$

For example, if Endpoint_A_ is found to have a 99% baseline correlation with Endpoint_B_, both endpoints are not individually adding information to the variance-covariance matrix, so only Endpoint_A_ should stay in the matrix and the value for η_B_ can be found with a scalar θ_BA_ multiplied by η_A_ (i.e., η_B_ = θ_BA_ η_A_). In another situation, if Endpoint_C_ is weakly correlated with Endpoint_D_, but not correlated at all with other endpoints, having many covariance parameters converging to 0 could cause stability problems. A solution would be to create a lone omega parameter, ω_C0_ (corresponding to individual parameter η_C0_) represent the uncorrelated part of Endpoint_C_ and address the correlation with Endpoint_D_ with a scalar θ_CD_ multiplied by η_D_ (i.e., η_C_ = η_C0_ + θ_CD_ η_D_). Actual examples were observed in model building and are discussed with the results.

Residual unexplained variability was modeled as additive for all endpoints, although proportional variability was explored it was not used. There were no transformations made to the observed values for most endpoints, except enzyme concentrations. Because the enzyme subscore for TIS is based on the enzyme with the highest value relative to the upper limit of normal (ULN), and this may change visit-to-visit, the values were modeled as the ratio of the observed value to the ULN for whichever enzyme is under observation. For an individual i at time j, the predicted ratio (R_ij, enz_ = End_enz, ij_) and additive RUV ($\epsilon$_ij, enz_) were treated as normalized by the ULN, and so were transformed to the denormalized scale for model fitting (Y_ij, enz_), as in Eq. (8).

8 $$\:Y_{ij,enz}=\left(R_{ij,enz}+\varepsilon_{ij,\;enz}\right)\cdot ULN_{enz}$$

Residual unexplained variability was fitted the “Uppsala way,” where the standard deviations of observations were fitted as fixed effects and the random error variance was fixed to 1, as is common when various error model structures may be explored. With this approach, technically only one residual error variance parameter is needed (as they are all fixed to 1), but in the present model all endpoints had a separate variance parameter. This was done because it was not expected to make a mechanical difference in the optimization routine and provided endpoint-specific epsilon shrinkage and conditional weighted residuals automatically while maintaining the freedom to explore other error models. Covariance of residuals could have been explored with a block sigma matrix, given the additive structure and the nature of the assessments occurring within the same visits but was not since baseline covariance was already considered in the model and the shared parameters already imply a degree of covariance.

There were insufficient data for extensive covariate modeling. The impact of MPDM on shared structural parameters and the baseline estimates for shared endpoints was explored. Since MPDM is a binary variable, the impact was assessed as a percentage change to the parameter values, respecting the constraints of each affected parameter. The selection of parameters on which to test covariate effects was guided by plausibility and diagnostics. Inclusion of covariate effects followed a stepwise approach, with forward-stepping $\alpha$ 0.01 and backward-stepping $\alpha$ 0.001.

## Clinical trial simulations

Clinical trials with a mixed representation of SPDM and MPDM patients could be simulated since the model could predict TIS response in SPDM. Clinical Trial Simulation (CTS) was used to assess the effect of continued dosing placebo or 600 mg every 4 weeks for 6 months in a group of 80% MPDM and 20% SPDM; this spread was intended to represent the 20% amyopathic DM patients in the general DM population [9], though the difference in SPDM and amyopathic DM is acknowledged. Other dosing levels were simulated but not shown here for proprietary reasons. The TIS response categories in 1000 trials of 120 patients per arm, stratified by DM type and pooled, were assessed.

In the simulated trials, parameter uncertainty was based on sampling the final Sampling Importance Resampling (SIR) iteration Box-Cox transformed variance-covariance matrix [16]. The simulation also added an empirical covariate effect to increase baseline MMT-8 in SPDM subjects to approach healthy normal. Simulated MMT-8 values greater than the maximum 150 were fixed to 150.

Given that TIS was the primary clinical endpoint for the trial and observed data for this endpoint were limited, TIS was the focus of simulations. Predictive checks can be examined to evaluate time courses for other endpoints.

### Software

The model was developed in NONMEM 7.5.0 [17] using First Order Conditional Estimation with Interaction (FOCE-I) and the ADVAN13 subroutine; given the block variance-covariance and large number of estimates, Stochastic Approximation Expectation-Maximization (SAEM) was considered, but it did not provide any stability or identifiability benefit over FOCE. Uncertainty of parameter estimates in the final model was assessed using SIR through Perl-speaks-NONMEM (PsN) 5.2.6 [18]; PsN was also used for a limited bootstrap to assess stability of the base model. Typical diagnostic plots were generated in R 4.0.3 [19]. Simulations to generate visual predictive checks (VPCs) and other output were performed using mrgsolve 0.10.4 [20]. Coefficient of variation for modified log-normal and logit-normal parameters were assessed using integration-based methods in pmxcv 0.0.1 [21]. Model code (with some details removed) is available in Supplementary Materials.

### Ethics statement

All study protocols were reviewed and approved by each clinical research site’s institutional review board or ethics committee and conducted in accordance with the Declaration of Helsinki and in compliance with all International Council for Harmonisation Good Clinical Practice Guidelines. All participants provided written, informed consent. The study was registered at ClinicalTrials.gov (NCT03181893).

## Results

### Study population

The demographics and some outcomes data are in Table 1. There were some demographics differences between the stages. CDASI-A was lower in Stage 3 (MPDM) than the other stages, and CFB for that endpoint was also lesser in magnitude. Randomization to each dose is not reported here for space but can be found on ClinicalTrials.gov if needed to aid interpretation.

**Table 1:** Demographics and outcome summaries

	Stage 1	Stage 2/2A	Stage 3	Total
DM Predominance	Skin	Skin	Muscle	All
Sample Size	32	25	18	75
*Demographics*				
Age (yr)	55.5 (25.0, 78.0)	52.0 (24.0, 71.0)	47.0 (21.0, 68.0)	52.0 (21.0, 78.0)
Sex	3 M, 29 F (9%, 91%)	1 M, 24 F (4%, 96%)	5 M, 13 F (28%, 72%)	9 M, 66 F (12%, 88%)
Race				
White	29 (91%)	24 (96%)	16 (89%)	69 (92%)
Asian	0	1 (4%)	1 (5.5%)	2 (3%)
Multiracial	1 (3%)	0	0	1 (1%)
Not Reported	2 (6%)	0	1 (5.5%)	3 (4%)
Weight (kg)	73.9 (53.8, 149.1)	80.2 (54.0, 123.1)	76.3 (50.0, 99.4)	76.7 (50.0, 149.1)
BMI (kg/m^2^)	27.3 (21.2, 43.4)	27.9 (19.0, 45.5)	27.7 (18.9, 37.6)	27.8 (18.9, 45.5)
Concomitant IVIG	4 Y, 28 N (12%, 88%)	5 Y, 20 N (20%, 80%)	6 Y, 12 N (33%, 67%)	15 Y, 60 N (20%, 80%)
Concomitant Corticosteroids	15 Y, 17 N (47%, 53%)	13 Y, 12 N (52%, 48%)	11 Y, 7 N (61%, 39%)	39 Y, 36 N (52%, 48%)
*Outcomes*				
Baseline CDASI-A	30.0 (18.0, 51.0)	33.0 (18.0, 68.0)	13.0 (1.0, 35.0)	27.0 (1.0, 68.0)
Week 12 CDASI-A	15.0 (4.0, 54.0)	13.0 (5.0, 51.0)	6.5 (0.0, 33.0)	11.0 (0.0, 54.0)
CFB CDASI-A	−13.5 (−41.0, 6.0)	−16.0 (−33.0, 5.0)	−3.5 (−22.0, 6.0)	−13.0 (−41.0, 6.0)
Week 12 TIS	NC	NC	45.0 (2.5, 92.5)	NC
TIS Category				
None	NC	NC	4 (22%)	NC
Minimal	NC	NC	14 (78%)	NC
Moderate	NC	NC	10 (56%)	NC
Major	NC	NC	7 (39%)	NC

Due to low sample sizes, continuous variables are represented as median and range; categorical variables are represented as the number of subjects in each grouping, with corresponding percentages shown aside these numbers. For TIS Category, percentages exceed 100% because categories represent exceeding a TIS threshold, so Moderate and Major are also Minimal, and Major is also Moderate. When values were not collected (NC), summaries including NC values were considered NC. Endpoint abbreviations are defined in Outcomes Assessments

Other Abbreviations: 2 A = 2 amended; BMI = body mass index; DM = dermatomyositis; F = female; IVIG = intravenous immune globulin; M = male; N = no; Y = yes

### Exposure-response model

The base ER model captured all the endpoints with good conditioning (Supplementary Table S1). Parameters with high uncertainty were those estimating E_max_ on endpoints with minimal response to dazukibart, which was an acceptable model result; several Emax values were also fixed to 0, 1, or −1 as the tests with these parameters unfixed were converging towards those extremes in a naive model without optimization (values from the naive model are in Supplementary Table S2). With the low number of MPDM participants, TIS-associated $\:{\omega\:}^{2}$ parameters had to be combined and some covariance addressed with fixed effects. Despite the high number of random effects, there were no issues with shrinkage greater than 25%. The $\:{\omega\:}^{2}$ on E_max, norm_ appears high (> 1), but since it is on a logit-transformed value the relative effect on %CV is low compared to log-transformation. The IIV for PhGA and TIS PhGA was shared and although the baseline parameters were similar, the model was more stable fitting them separately. The model was moderately stable, with 72% successfully completing on a limited bootstrap (*N* = 200; stratified on trial stage), with runs failing typically due to rounding errors.

The independent IIV parameter for ExGA was the only one not included in the block $\:\varOmega\:$ matrix. Correlation to the other endpoints was captured indirectly by the shared rate and effect parameters, and the part of PhGA IIV used to describe ExGA IIV, as in Eq. (9), which provides an example of the application of Eq. (7).

9 $$\:{\eta\:}_{ExGA}^{\mathrm{*}}={\eta\:}_{ExGA}+{\theta\:}_{\mathrm{X}PhGA,ExGA}\cdot\:{\eta\:}_{PhGA}$$

**Fig. 2:**
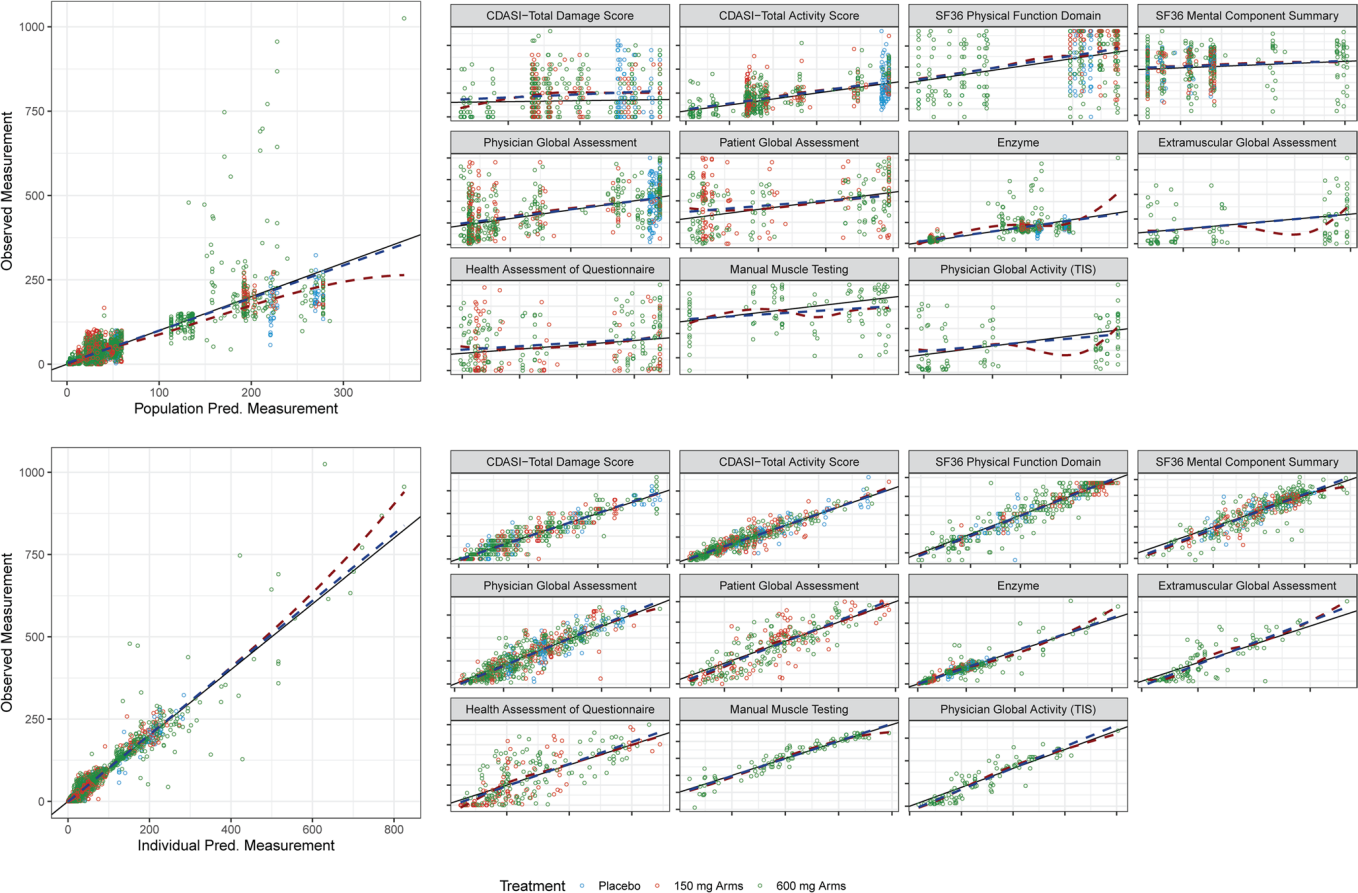
Prediction diagnostic plots for final model. For each set of predictions, endpoints are faceted out for focused assessment, but all endpoints are also pooled into an overall plot on the left, to give an impression of the full range fit by the model. Observations are colored based on the dose the participant was receiving at the time. Axis values are not provided for each endpoint, only the overall plots, to provide more space to compare distribution along the line of unity (black, solid). The blue dashed line is the best fit linear regression, and red dashed is the best fit smooth spline. Endpoint abbreviations are defined in Outcomes Assessments

The final model had covariate effects on CDASI-A and SF-36 PFD baselines and on E_plac_. Thus, the final model primarily improved fit for MPDM subjects, which would be expected to improve TIS predictions. The parameters with SIR-derived uncertainty are in Table 2 (covariance parameters are in Supplementary Table S3), and diagnostic figures can be found in Fig. 2. Notably, in SIR all unfixed E_max, m_ values excluded 0 from the confidence intervals even though these were included in the testable range; if this did not result from an unknown error in the SIR algorithm, it supports some degree of efficacy in each endpoint. Conversely, the confidence intervals do not exclude 100% (or 1) for the placebo parameter, indicating the typical SPDM patient may not experience a placebo response.

**Table 2:** Final model parameter estimates

Parameter	Value	RSE	SIR		SHR
			Median	95% CI	
Objective Function Value	20061.6				
Condition Number	109.6				
Turnover rate for endpoints,$\:\theta\:$_KOUT_ (wk^− 1^)	0.246	6.66	0.246	(0.219, 0.274)	
E_max, norm_,$\:\theta\:$_EMAXNORM_ (%)	56.4	7.36	56.3	(49.3, 64.1)	
Placebo non-steady state (E_pla_),$\:\theta\:$_PBO_ (%)	103	1.77	103	(99.8, 106)	
MPDM on E_pla_,$\:\theta\:$_COVS3ALLPBO_ (%)	6.5	51.1	6.52	(1.38, 11.9)	
*Baselines*					
CDASI-D baseline,$\:\theta\:$_CDASIDBASE_	3.62	11	3.61	(3, 4.33)	
CDASI-A baseline,$\:\theta\:$_CDASIABASE_	28.3	5.07	28.3	(26.2, 30.9)	
MPDM on baseline CDASI-A,$\:\theta\:$_COVS3CDASIA_ (%)	−51.8	8.21	−51.9	(−59.7, −44.6)	
SF-36 PFD baseline,$\:\theta\:$_SF36PFDBASE_	43.8	3.33	43.8	(41.4, 46.3)	
MPDM on baseline SF-36 PFD,$\:\theta\:$_COVS3PDF_ (%)	−24.3	19.5	−23.8	(−31.5, −16.6)	
SF-36 MCS baseline,$\:\theta\:$_SF36MCSBASE_	48.2	2.25	48.2	(46.2, 50)	
PhGA baseline,$\:\theta\:$_PhGABASE_	54.7	3.8	54.7	(51.5, 57.9)	
PtGA baseline,$\:\theta\:$_PtGABASE_	59.4	6.18	59.4	(53.1, 65.1)	
Enzymes baseline,$\:\theta\:$_EnzymeBASE_ (xULN)	1.19	5.6	1.19	(1.07, 1.32)	
ExGA baseline,$\:\theta\:$_ExGABASE_	2.97	11.1	2.96	(2.46, 3.5)	
HAQ baseline,$\:\theta\:$_HAQBASE_	37.2	9.61	37.2	(31.3, 43)	
MMT8 baseline,$\:\theta\:$_MMT8BASE_	113	3.71	113	(107, 121)	
TIS PhGA baseline,$\:\theta\:$_TISPhGABASE_	4.83	5.56	4.82	(4.39, 5.27)	
*E*_*max*_ *estimates*					
E_max_ on CDASI-D,$\:\theta\:$_CDASIDEMAX_ (%)	−12.1	41	−12.2	(−19.6, −4.58)	
E_max_ on CDASI-A,$\:\theta\:$_CDASIAEMAX_ (%)	−100	Fixed			
E_max_ on SF-36 PFD,$\:\theta\:$_SF36PFDEMAX_ (%)	7.35	42.4	7.07	(1.64, 12.8)	
E_max_ on SF-36 MCS,$\:\theta\:$_SF36MCSEMAX_ (%)	0	Fixed			
E_max_ on PhGA,$\:\theta\:$_PhGAEMAX_ (%)	−100	Fixed			
E_max_ on PtGA,$\:\theta\:$_PtGAEMAX_ (%)	−79.7	6.99	−79.8	(−89.4, −70.5)	
E_max_ on Enzymes,$\:\theta\:$_EnzymeEMAX_ (%)	−51.1	8.41	−51.2	(−59, −42.2)	
E_max_ on ExGA,$\:\theta\:$_ExGAEMAX_ (%)	−100	Fixed			
E_max_ on HAQ,$\:\theta\:$_HAQEMAX_ (%)	−73.6	8.73	−73.6	(−86.9, −61.6)	
E_max_ on MMT8,$\:\theta\:$_MMT8EMAX_ (%)	17.6	22.1	17.7	(10.6, 23.9)	
E_max_ on TIS PhGA,$\:\theta\:$_TISPhGAEMAX_ (%)	−100	Fixed			
*Additive Residual Unexplained Variability (standard deviations)*					
Additive RUV for CDASI-D,$\:\theta\:$_CDASIDRUV_	1.43	3.21	1.43	(1.35, 1.51)	6.28
Additive RUV for CDASI-A,$\:\theta\:$_CDASIARUV_	4.67	3.37	4.68	(4.44, 4.95)	7.96
Additive RUV for SF-36 PFD,$\:\theta\:$_SF36PFDRUV_	4.68	4.25	4.67	(4.34, 5.04)	11.7
Additive RUV for SF-36 MCS,$\:\theta\:$_SF36MCSRUV_	5.78	4.28	5.78	(5.35, 6.22)	10.6
Additive RUV for PhGA,$\:\theta\:$_PhGARUV_	12	3.21	12	(11.3, 12.7)	5.63
Additive RUV for PtGA,$\:\theta\:$_PtGARUV_	15.4	4.92	15.4	(14, 16.7)	5.7
Additive RUV for Enzymes,$\:\theta\:$_EnzymeRUV_	0.352	3.49	0.352	(0.33, 0.376)	6.86
Additive RUV for ExGA,$\:\theta\:$_ExGARUV_	1.2	7.89	1.2	(1.07, 1.37)	6.36
Additive RUV for HAQ,$\:\theta\:$_HAQRUV_	19	5.19	19	(17.5, 20.7)	2.86
Additive RUV for MMT8,$\:\theta\:$_MMT8RUV_	7.32	10	7.28	(6.09, 8.57)	11.9
Additive RUV for TIS PhGA,$\:\theta\:$_TISPhGARUV_	1.06	8.5	1.06	(0.912, 1.23)	4.68
*Interindividual Variability*					
IIV on E_max, norm_,$\:{\omega\:}^{2}$_IIVEMAXNORM_	1.68 (45%)	25.2	1.68	(1.06, 2.56)	20.9
IIV on E_pla_,$\:{\omega\:}^{2}$_IIVPBO_	0.00843 (9%)	26.3	0.00854	(0.00498, 0.0127)	18.4
IIV on baseline CDASI-D,$\:{\omega\:}^{2}$_IIVCDASID_	0.564 (104%)	18.4	0.563	(0.43, 0.765)	4.85
IIV on baseline CDASI-A,$\:{\omega\:}^{2}$_IIVCDASIA_	0.147 (41%)	19.1	0.149	(0.112, 0.191)	4.54
IIV on baseline SF-36 PFD,$\:{\omega\:}^{2}$_IIVSF36PFD_	0.0589 (25%)	17.8	0.0592	(0.0456, 0.0775)	2.57
IIV on baseline SF-36 MCS,$\:{\omega\:}^{2}$_IIVSF36MCS_	0.0311 (18%)	19.2	0.0314	(0.0232, 0.0418)	5.05
IIV on baseline PhGA,$\:{\omega\:}^{2}$_IIVPhGA_	0.0904 (31%/37%)	18.9	0.091	(0.0683, 0.118)	6.36
IIV on baseline PtGA,$\:{\omega\:}^{2}$_IIVPtGA_	0.111 (35%)	29	0.111	(0.0743, 0.158)	2.64
IIV on baseline Enzymes,$\:{\omega\:}^{2}$_IIVEnzyme_	0.0591 (44%)	17.9	0.0594	(0.0458, 0.074)	5.29
IIV on baseline HAQ/MMT8,$\:{\omega\:}^{2}$_IIVHAQMMT8_	0.24 (53%/52%)	31.6	0.244	(0.158, 0.366)	2.89
IIV on baseline ExGA,$\:{\omega\:}^{2}$_IIVExGA_	0.0592 (33%)	56.2	0.0588	(0.0214, 0.117)	20.6
MMT8$\:{\omega\:}^{2}$from HAQ,$\:\theta\:$_PROHAQMMT_	−0.378	14.2	−0.38	(−0.472, −0.286)	
Part ExGA$\:{\omega\:}^{2}$from PhGA,$\:\theta\:$_PROExGAPhGA_	1	Fixed			

Covariance parameter estimates for baseline interindividual variability (IIV) values are in Supplementary Table S3 for space. For IIV parameters, the variance estimate is shown, with %CV of the resulting structural parameter in parentheses; when multiple structural parameters are described with a single parameter, the %CV for each are shown in order of displayed fixed effect, separated by slashes (for example, PhGA IIV %CV is shown as “PhGA %CV/TIS PhGA %CV”). Endpoint abbreviations are defined in Outcomes Assessments. RUV was modeled with fixed effect estimates for the standard deviations, so those estimates are shown before the random effect parameter estimates; the associated random effect parameters (sigmas) were all fixed to 1, so only the shrinkage parameter (based on standard deviation of weighted residuals) is shown with the corresponding RUV estimate

Other Abbreviations: CI = confidence interval; CV = coefficient of variation; MPDM = muscle-predominant dermatomyositis; RSE = relative standard error; RUV = residual unexplained variability; SHR = shrinkage; SIR = sampling importance resampling

**Fig. 3:**
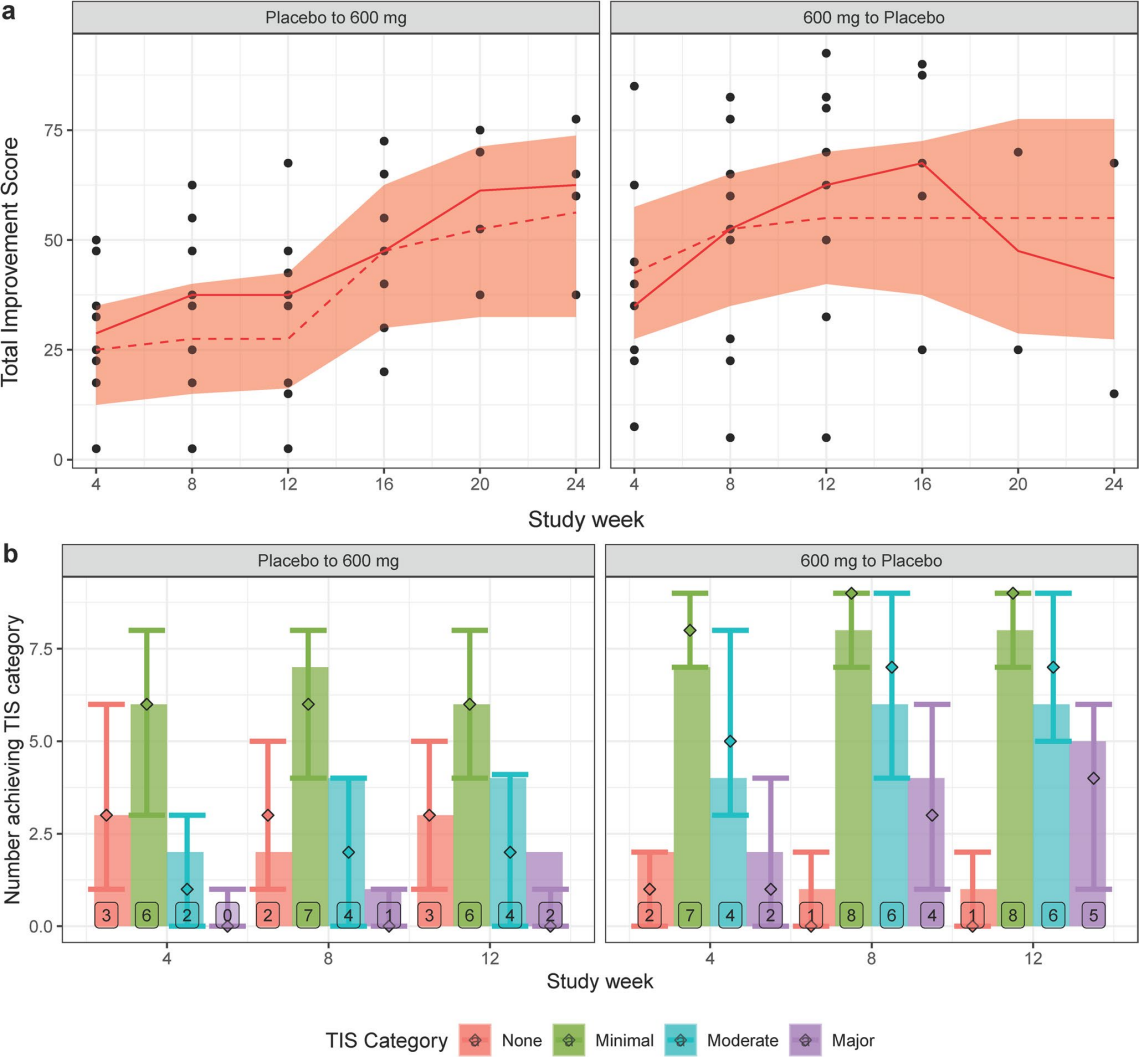
Visual predictive checks for total improvement score. Both plots are stratified by crossover arm. The plots in (a) are continuous TIS scores through end-of-study, with observed individual scores in black and median in red; medians and 95% confidence intervals from 1000 simulated trials are shown as dashed lines and bands. Only median is used for the predictive checks in (a) because of the small sample size. The plots in (b) are TIS responder categories up to primary time (12 weeks), with bars and text indicating number in category, and point and error bars indicating median and 95% distribution of responders in simulated trials

**Fig. 4:**
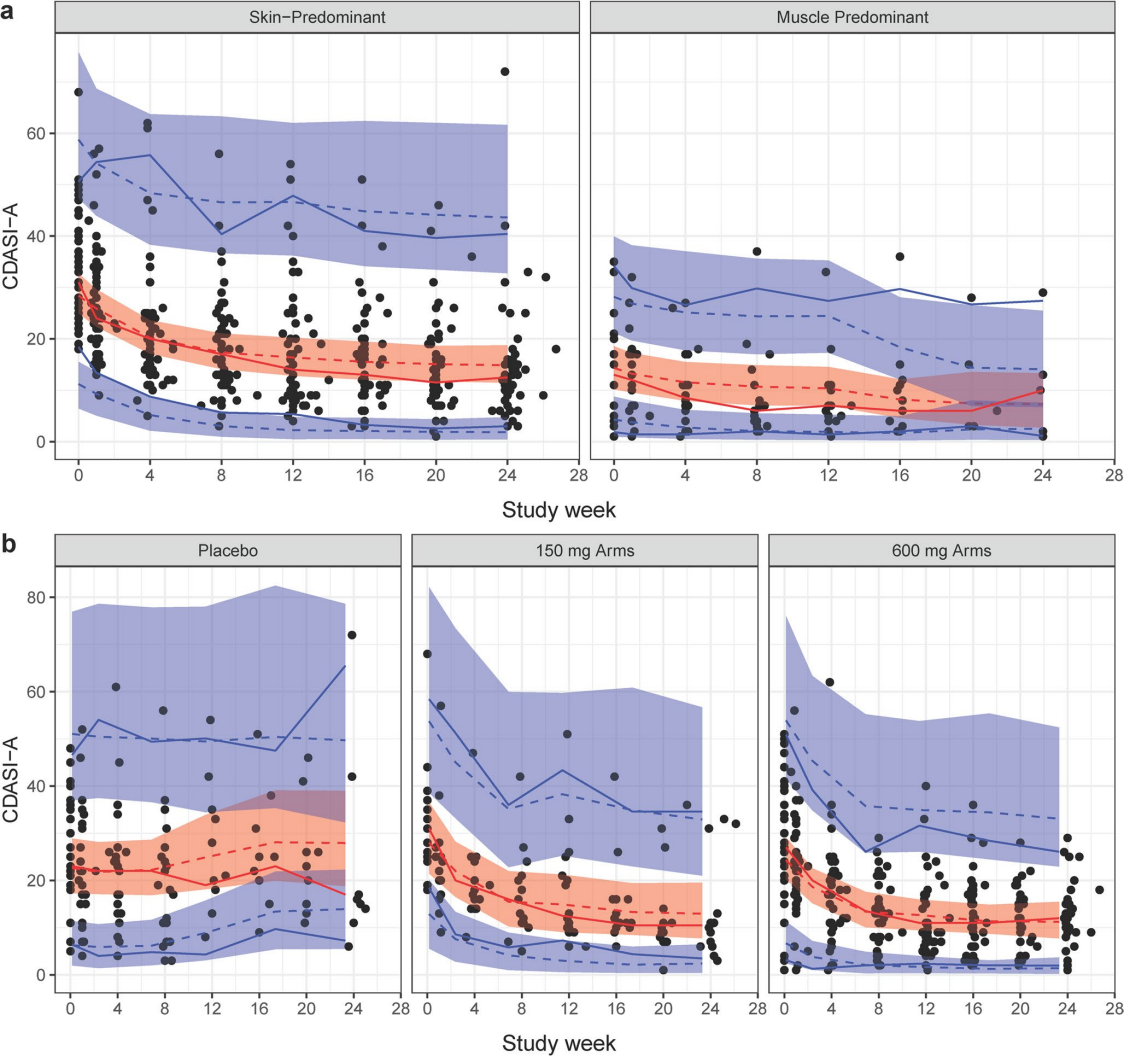
Visual predictive checks for cutaneous dermatomyositis disease area and severity index. Both plots show observed individual CDASI Activity scores in black, 95% distribution in blue, and median in red; 95% confidence intervals and medians for these quantiles from 1000 simulated trials are shown as bands and dashed lines. The plots are stratified by dermatomyositis type (a) or dosing level (b)

The model was found to be adequately predictive of clinical responses. TIS was predicted well in terms of continuous score and the TIS responder categories, as shown in Fig. 3. Timecourses for CDASI-A response was differentiated for SPDM and MPDM patients (Fig. 4), with some minor discrepancy in the MPDM group as sample size decreased in later timepoints. Predictive capacity in other endpoints can be seen in Figures S1-S2.

### Simulations

**Fig. 5:**
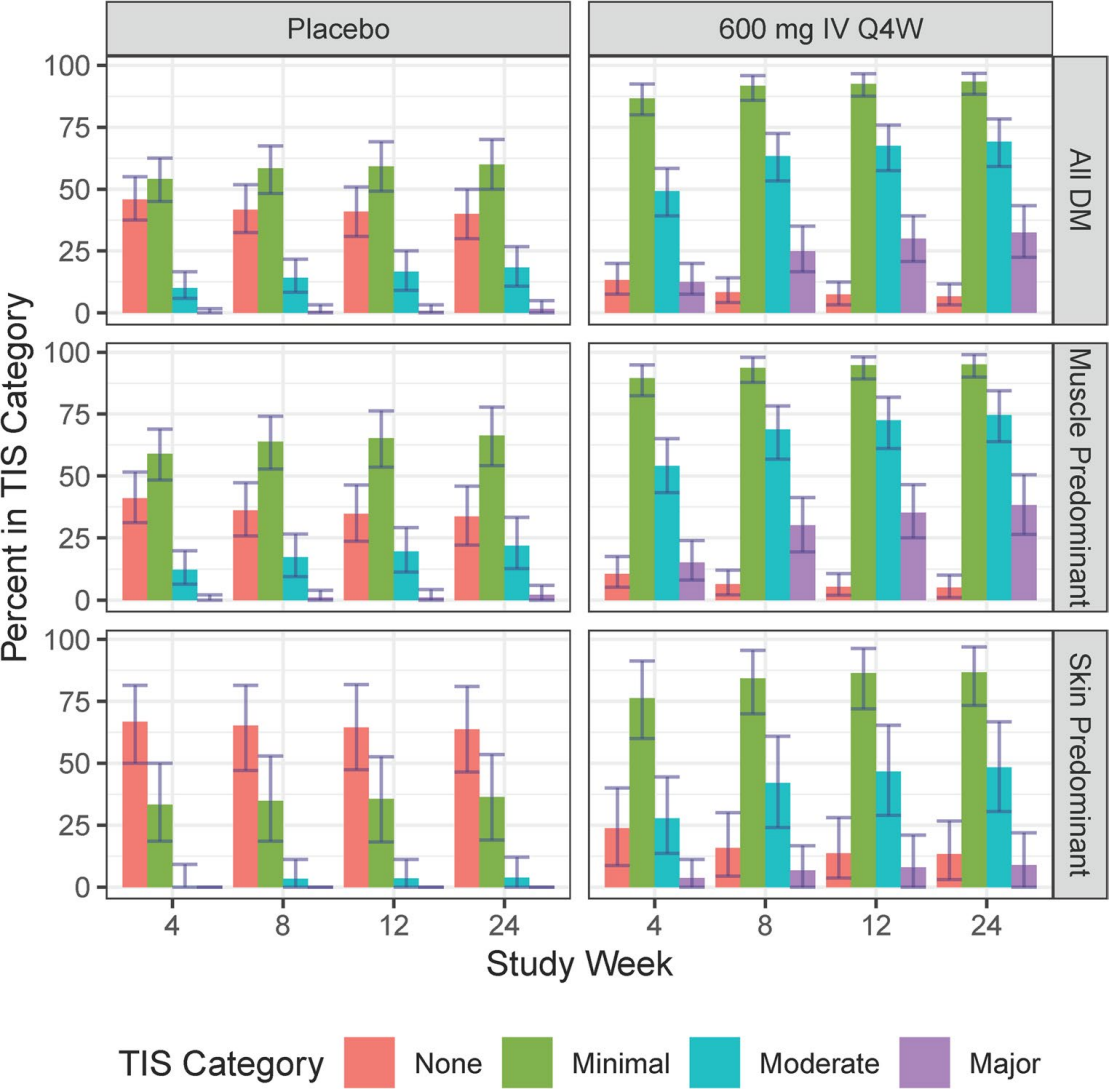
Total Improvement Score predictions in simulated clinical trials. Bars and error bars are the median and 90% prediction interval for the proportion of subjects achieving the labeled TIS categories. Other Abbreviations: DM = dermatomyositis; IV = intravenously; Q4W = every four weeks

The CTS results are shown in Fig. 5. The model predicts that the 600 mg dose will have a greater effect than placebo in both SPDM and MPDM patients. In both placebo and active simulated patients, TIS response was more pronounced in MPDM. Major response rate increased over time whereas Minor response plateaued relatively quickly.

## Discussion

An exposure-response model was developed to describe multiple outcomes in DM in response to dazukibart or placebo. The model is built on the assumption that each endpoint is controlled by a shared underlying process inhibited by the binding of IFN_β_, which is mechanistically plausible and have shown to adequately describe the data. The assumption allows a certain degree of extrapolation into the endpoints with limited information and informed dose selection in trials where those endpoints will be primary. The approach also establishes a strong example of large-scale joint modeling where the breadth of data can supplement a lack of depth in each observed clinical endpoint.

The joint modeling approach is most valuable in the ability to predict unmeasured endpoints. For example, MMT-8, a muscle endpoint, was only collected for MPDM, and HAQ was collected in some SPDM patients. Theoretically, because HAQ shares a random effect estimate with MMT-8, the covariance of skin endpoints with HAQ would therefore inform the missing MMT-8 in SPDM. As a result, MMT-8 in SPDM patients were predicted to be lower than what was observed in MPDM patients (Figure S3), which was also consistent with clinical understanding. In the future, multivariate distribution can be further improved by robust clinical data across the DM spectrum.

While the simulation predicting TIS response in SPDM patients is founded in the structure of the model and may be valid if correlations and shared parameters are generalizable, the assumption that TIS in this group can be predicted without any observation is a strong one. If correlation between CDASI-A and ExGA or MMT-8 is different in this group (which is not necessarily unlikely), then the predictions from the simulation are not valid. Absent TIS data focused on response in SPDM patients, the model provides some guidance for expected response; this predicted response however should be viewed as a modestly informed guess rather than an accurate backfill of missing data not collected in the trial.

External data were evaluated to assess the predictions of TIS response in SPDM patients, although this evaluation was ad hoc and informal. *Posthoc* analyses of the large Phase 3 trials for IVIG [22] and lenabasum [23] in DM patients stratified response by CDASI-A and MMT-8 baselines. Specifically, the IVIG analysis showed that MPDM patients exhibited a higher TIS response and lower CDASI-A at baseline, whereas the lenabasum analysis showed that SPDM patients exhibited a lower TIS response and a perfect MMT-8 score at baseline (Fig. 5 may aid this comparison). The placebo responses in those trials also track well with the overall placebo response predicted by the model: the lenabasum Phase 3 placebo (*N* = 71) presented a Week 28 TIS of 27.2, for IVIG the placebo (*N* = 48) TIS as Week 16 was 21.7, both of which are consistent for the present model placebo TIS prediction of ~ 25 ± 10 from Weeks 4–24 (Figs. 3 and 5). These results were generally consistent with the model predictions. The ER model can be further refined to facilitate between-trial comparisons using model-based meta-analysis. A formal investigation especially in SPDM patients would be especially valuable in this disease state.

The modeling approach stems from applications of item response theory (IRT) to pharmacometrics [24], in which each endpoint is dependent on a drug-dependent latent variable. In the present model, the drug-dependent variable (fraction of IFN_β_bound) was neither latent nor infinite but instead was an observable pharmacological index that associates with downstream biomarkers. The approach further deviates from IRT by not treating endpoints as composite outcomes with categorical or bounded subscores so the typical item characteristic function parameters can be specified. Nevertheless, the use of separate E_max_ parameters for each endpoint, combined with the effect of modeling observed baseline as an altered steady state, did result in describing the differential response characteristics similarly described by IRT. Simulations generated from this model can be used to build priors for a new model that accurately reflects the numeric properties of the scores fitted. A TIS-specific IRT model dependent upon some IFN_β_ -driven latent variable would be ideal, and a Bayesian approach to the sparsely sampled but extensive Phase 3 data would be a robust option. There are additional latent variable-based modeling approaches that this model shares some elements with, such as the indirect latent variable response model (ILVRM) [25]. This approach similarly treats a dynamic, IRM-style latent variable as the driver for an arbitrary number of outcomes, and may be considered an alternative approach to what was done here. Like with IRT, the fact that the “latent variable” in the present model is actually a pharmacodynamic marker is one major difference from ILVRM, and that continuous endpoints were used exclusively. An approach predicting multiple continuous endpoints driven by a latent variable was published by Goteti et al. [26] after the present modeling work was done serves as an example of an alternative approach to this model.

There are several limitations in the assumptions and methodology for this analysis. One critical limitation was that parameters could be shared. The shared parameters k_out_, E_max, norm_ and E_plac_ were useful in fitting many endpoints without merely combining many models, but realistically each endpoint would deviate from these shared values to some degree. Most notably, the major TIS achievers in placebo subjects was outside the prediction interval for the model even after accounting for E_plac_ differences in MPDM, suggesting some of the larger contributors to TIS (MMT-8 or the global assessments) may have greater E_plac_ than the other endpoints.

Another potential limitation of the model is the assumption that baseline observations could be treated as correlated. However, since correlations were fitted, no correlation (except when IIV estimates were shared) was assumed to exist. The covariance matrix ultimately served to provide more stability to the model and to generate more valid simulated populations. On a large sample level, the summary results would not be as sensitive to the correlations since the result would be indistinguishable from that where all covariance terms are zero. The covariance matrix ensures each simulated subject is more representative of a real patient.

A minor, but noteworthy limitation is that the numeric and distributional properties of outcome measures were largely ignored in model fitting. That is, each endpoint in this analysis was fitted as continuous, correlated and on the identity scale. Although each endpoint is bound and some are discrete, these numerical properties were not considered given the complexity of the model and the low number of subjects with some endpoints, due to concerns that the required likelihood-based modeling would introduce more instability to the model. However, there are an abundance of statistical methods to properly fit these data while respecting their numerical properties and may have allowed for some simplification of the overall structure, such as beta regression [27] and bounded integer modeling [28]. A more robust analysis would ideally reflect a true distribution of these outcome measures without applying the assumptions of the continuous, normal distribution. A potential bias introduced to the model by not considering the bounds was the estimate of k_out_, which may have been impacted by data reaching an apparent plateau, which was just an upper or lower bound (as with MMT-8).

Overall, the complexity of the model is valuable and unique to the dazukibart DM data, which were sparse and limited. Considerations to use a relatively more complex model are 3-pronged. (1) Simpler models are beneficial when there are abundant data for a singular, focused, validated endpoint, and certainly provide a robust framework for understanding exposure-response in that setting; complex models, despite also being useful in that setting, are often necessary when data are sparser and more diverse, and the decisions being made from these data are not self-evident. (2) The complex model allows extrapolation without excessive justification or assumptions for response at untested doses (assessed but not shown here) and in participants with unmeasured endpoints of interest, whose response may be critical to the success of future trials [22, 23]. (3) When modeling derived outcomes, it is almost always better to model as much of the directly observed data used in the derivation as possible, even if it impacts apparent prediction quality of the derived data [29]. Therefore, a shift in derived predictions relative to derived observations may indicate that the model is more informative than the observed results alone (provided conventional predictive check quality criteria are met).

## Conclusion

The model developed in this analysis is a valuable tool for predicting the response of DM patients to dazukibart and may have broader applicability to patients with other IIMs. The methodology applied in this approach is novel and borrows concepts from IRT (among other increasingly popular techniques), maintaining adequate stability, predictive capacity and flexibility. In the specific application, more work can be done to refine or learn from the model to develop a robust, but simpler approach to use in future trials. In general, this approach should be tested in other rare diseases where multiple endpoints are measured but data are limited.

## Supplementary Information

Below is the link to the electronic supplementary material.

Supplementary File 1 (MOD 14.8 KB)

Supplementary File 2 (DOCX 30.8 KB)

Supplementary File 3 (DOCX 1.44 MB)

## Data Availability

Upon request, and subject to review, Pfizer will provide the data that support the findings of this study. Subject to certain criteria, conditions and exceptions, Pfizer may also provide access to the related individual de-identified participant data. See https://www.pfizer.com/science/clinical-trials/trial-data-and-results for more information.

## Footnotes

1 The “DM” label for baseline values is to distinguish from the IFN_β_-corrected baseline discussed later; the “DM” baseline is the value that should align with observed baselines.

## Declarations

### Statements and declarations

All authors are current or former employees of Pfizer and own stock in the company. Pfizer supported the study and the reported analysis.
