# Supplementary material for: Early exposure-response modeling of an interferon-beta monoclonal antibody (dazukibart) in adults with dermatomyositis
Source: J Pharmacokinet Pharmacodyn. 2026 Mar 24;53(3):17. doi: 10.1007/s10928-026-10022-1 (PMC13013141; doi:10.1007/s10928-026-10022-1)
Supplement: Supplementary file 2 — (DOCX 30.8 KB) [file 10928_2026_10022_MOESM2_ESM.docx]

Supplementary Material: Dazukibart Pharmacokinetic/Pharmacodynamic Model

John P. Prybylski, Jing ‘Daisy’ Zhu, Christopher Banfield, Arnab Mukherjee, Vivek Purohit

The PK model for dazukibart was fitted using healthy participant (NCT02766621) and dermatomyositis patient data (present study). Some healthy participants were given subcutaneous dazukibart, so a depot compartment and related absorption parameters were included in the population PK model. The model was developed with minimal covariates (only fixed allometric constants). The selected model was typical structural model for monoclonal antibodies and is composed of two compartments with first-order subcutaneous absorption [1]. There was no evidence of target-mediated drug disposition or other effects of pharmacodynamic targets on pharmacokinetics, so the individual PK parameters were used in sequential PK/PD modeling [2].

The PD base model attempted to semi-mechanistically describe the binding of IFN${}_{\beta}$. The system describing the model is displayed in the equations below. The binding and elimination of IFN${}_{\beta}$ uses a quasi-steady state approximation, and the total drug concentration was treated as equivalent to free drug concentration. IFN${}_{\beta}$ homeostasis was represented with a simple turnover model. The central concentration of dazukibart ($C$) was used in combination with the quasi-steady state binding constant ($K_{SS}$) to estimate the fraction of total IFN${}_{\beta}$ that was bound. The total drug concentration $C$ was considered entirely free in the approximation because of the low molar ratio of target to drug (<< 1). Bound and unbound IFN${}_{\beta}$ were eliminated by internalization (with rate constant $k_{int}$) and innate degradation (with rate constant $k_{deg}$), respectively, and free/unbound IFN${}_{\beta}$ was synthesized at a rate $k_{syn}$, which was determined from a steady-state assumption using baseline IFN${}_{\beta}$. Because many IFN${}_{\beta}$ concentrations were below the limit of quantification, the model was fit to the data using a likelihood-based approach that accounted for censored data (M3) [3].

$$\text{IFN}_{\text{total}}=\text{IFN}_{\text{bound}}+\text{IFN}_{\text{free}}$$

$$\text{IFN}_{\text{bound}}=\text{IFN}_{\text{total}}\cdot\frac{C}{C+K_{SS}}$$

$$\frac{d\text{IFN}_{\text{total}}}{d\text{t}}=k_{syn}-k_{deg}\cdot\text{IFN}_{\text{free}}-k_{int}\cdot\text{IFN}_{\text{bound}}$$

The model also incorporated additional downstream biomarkers, but the depth of that analysis is outside the scope of the present work. The final model parameter estimates for the PK/PD (PD limited to IFN${}_{\beta}$) are presented in the table below.

## PK/PD model parameter estimates.

| Parameter | Value | RSE | SIR | |  | SHR |
| --- | --- | --- | --- | --- | --- | --- |
|  |  |  | Median | 95% CI | |  |
| *PK Parameters* |  |  |  |  | |  |
| Systemic Clearance, $\theta$_CL_ (L/hr) | 0.00673 | 2.93 | 0.00673 | (0.00635, 0.00708) | |  |
| Baseline Weight (kg) on CL | 0.75 | Fixed |  |  | |  |
| Central Volume, $\theta$_VC_ (L) | 3.05 | 3.34 | 3.06 | (2.86, 3.25) | |  |
| Baseline Weight (kg) on VC | 1 | Fixed |  |  | |  |
| Intercompartmental Clearance, $\theta$_Q_ (L/hr) | 0.0196 | 8.36 | 0.0196 | (0.0166, 0.0229) | |  |
| Baseline Weight (kg) on Q | 0.75 | Fixed |  |  | |  |
| Peripheral Volume, $\theta$_VP_ (L) | 2.55 | 2.87 | 2.55 | (2.4, 2.69) | |  |
| Baseline Weight (kg) on VP | 1 | Fixed |  |  | |  |
| Subcutaneous Absorption Rate Constant, $\theta$_KA_ (hr^-1^) | 0.0106 | 21.5 | 0.0108 | (0.00743, 0.0155) | |  |
| Subcutaneous Bioavailability, $\theta$_BIOAV_ (%) | 73.1 | 5.25 | 73.2 | (66.2, 80.9) | |  |
| Subcutaneous Absorption Lag-time, $\theta$_ALAG_ (hr) | 0.933 | 5.01 | 0.929 | (0.844, 0.982) | |  |
| Proportional RUV, $\theta$_RUVPRO_ (%CV) | 19.7 | 1.94 | 19.7 | (19, 20.5) | | 1.12 |
| Additive RUV, $\theta$_RUVADD_ (ng/mL) | 133 | 16.6 | 134 | (94.9, 181) | |  |
| *PD Parameters* |  |  |  |  | |  |
| IFN${}_{\beta}$ Baseline, $\theta$_IFNBASE_ (pg/mL) | 0.0133 | 56.5 | 0.0132 | (0.00591, 0.0266) | |  |
| DM Mulitplier to IFN${}_{\beta}$, $\theta$_DMIFNBASE_ (fold) | 34.5 | 11.2 | 35 | (17.1, 72.4) | |  |
| IFN${}_{\beta}$ Degredation Rate Constant, $\theta$_IFNKDEG_ (d^-1^) | 211 | 77.2 | 209 | (76.8, 598) | |  |
| Quasi-steady state Binding Constant, $\theta$_KSS_ (pM) | 80.2 | 56.8 | 80.7 | (33.9, 170) | |  |
| Bound IFN${}_{\beta}$ Elimination Rate Constant, $\theta$_IFNKINT_ (d^-1^) | 0.774 | 58.3 | 0.757 | (0.329, 1.97) | |  |
| IFN${}_{\beta}$ Proportional RUV, $\theta$_IFNRUVPRO_ (%CV) | 86.7 | 7.02 | 87.3 | (76.8, 101) | | 2.46 |
| *Variance-Covariance* |  |  |  |  | |  |
| IIV on CL, $\omega^{2}$_IIVCL_ (%CV) | 29.4 | 14.2 | 29.4 | (25.6, 33.9) | | 11.2 |
| IIV on VC, $\omega^{2}$_IIVVC_ (%CV) | 32 | 15.9 | 32.1 | (27.4, 37.5) | | 14.2 |
| CL, VC Covariance, $\omega_{cov}$_CL.VC_ | 0.0416 | 25 | 0.0408 | (0.025, 0.0617) | |  |
| IIV on KA, $\omega^{2}$_IIVKA_ (%CV) | 81.6 | 43.7 | 84.4 | (54, 145) | | -0.912 |
| IIV on IFN${}_{\beta}$ baseline, $\omega^{2}$_IFN_ (%CV) | 66.5 | 35.2 | 67.6 | (47.2, 97.2) | | 19.1 |

All IIV parameters are shown as %CV from the transformation $\sqrt{e^{\omega^{2}}-1}$ because the variability is modeled as lognormal. Shrinkage for KA IIV was calculated using only empirical Bayes estimates that were non-zero, given the small number of participants given drug subcutaneously, and was estimated as slightly negative (standard deviation of individual estimates slightly larger than the standard deviation form of the fitted IIV estimate). RUV was modeled with fixed effect estimates for the standard deviations, so those estimates are shown before the random effect parameter estimates; the associated random effect parameters (sigmas) were all fixed to 1, so only the shrinkage parameter (based on standard deviation of weighted residuals) is shown with the corresponding RUV estimate.

Other Abbreviations: CI = confidence interval; CV = coefficient of variation; DM = dermatomyositis; IIV = interindividual variability; PK = pharmacokinetics; RSE = relative standard error; RUV = residual unexplained variability; SHR = shrinkage; SIR = sampling importance resampling.

## References

1. Ryman JT, Meibohm B. Pharmacokinetics of monoclonal antibodies. *CPT: Pharmacometrics & Systems Pharmacology*. 2017;6(9):576-588.

2. Zhang L, Beal SL, Sheiner LB. Simultaneous vs. Sequential analysis for population PK/PD data I: Best-case performance. *Journal of Pharmacokinetics and Pharmacodynamics*. 2003;30(6):387-404. doi:[10.1023/B:JOPA.0000012998.04442.1f](https://doi.org/10.1023/B:JOPA.0000012998.04442.1f)

3. Beal SL. Ways to fit a PK model with some data below the quantification limit. *Journal of Pharmacokinetics and Pharmacodynamics*. 2001;28(5):481-504.
