# Supplementary material for: Early exposure-response modeling of an interferon-beta monoclonal antibody (dazukibart) in adults with dermatomyositis
Source: J Pharmacokinet Pharmacodyn. 2026 Mar 24;53(3):17. doi: 10.1007/s10928-026-10022-1 (PMC13013141; doi:10.1007/s10928-026-10022-1)
Supplement: Supplementary file 3 — (DOCX 1.44 MB) [file 10928_2026_10022_MOESM3_ESM.docx]

### Supplementary Table S1. Base model parameter estimates.

| Parameter | Value | RSE | SHR |
| --- | --- | --- | --- |
| Objective Function Value | 20128 |  |  |
| Condition Number | 75.2 |  |  |
| Turnover rate for endpoints, $\theta$_KOUT_ (wk^-1^) | 0.245 | 6.77 |  |
| E_max,norm_, $\theta$_EMAXNORM_ (%) | 56.2 | 7.07 |  |
| Placebo non-steady state (E_pla_), $\theta$_PBO_ (%) | 105 | 1.58 |  |
| *Baselines* |  |  |  |
| CDASI-D baseline, $\theta$_CDASIDBASE_ | 3.6 | 12 |  |
| CDASI-A baseline, $\theta$_CDASIABASE_ | 24 | 6.49 |  |
| SF-36 PFD baseline, $\theta$_SF36PFDBASE_ | 41 | 3.33 |  |
| SF-36 MCS baseline, $\theta$_SF36MCSBASE_ | 48.1 | 2.3 |  |
| PhGA baseline, $\theta$_PhGABASE_ | 54.7 | 3.97 |  |
| PtGA baseline, $\theta$_PtGABASE_ | 58.6 | 6.94 |  |
| Enzymes baseline, $\theta$_EnzymeBASE_ (xULN) | 1.19 | 5.65 |  |
| ExGA baseline, $\theta$_ExGABASE_ | 3.06 | 11.6 |  |
| HAQ baseline, $\theta$_HAQBASE_ | 34.6 | 10.5 |  |
| MMT8 baseline, $\theta$_MMT8BASE_ | 118 | 4.18 |  |
| TIS PhGA baseline, $\theta$_TISPhGABASE_ | 4.95 | 5.78 |  |
| *E_max_ estimates* |  |  |  |
| E_max_ on CDASI-D, $\theta$_CDASIDEMAX_ (%) | -11.5 | 45.9 |  |
| E_max_ on CDASI-A, $\theta$_CDASIAEMAX_ (%) | -100 | Fixed |  |
| E_max_ on SF-36 PFD, $\theta$_SF36PFDEMAX_ (%) | 6.46 | 50.4 |  |
| E_max_ on SF-36 MCS, $\theta$_SF36MCSEMAX_ (%) | 0 | Fixed |  |
| E_max_ on PhGA, $\theta$_PhGAEMAX_ (%) | -100 | Fixed |  |
| E_max_ on PtGA, $\theta$_PtGAEMAX_ (%) | -80.6 | 6.65 |  |
| E_max_ on Enzymes, $\theta$_EnzymeEMAX_ (%) | -50.2 | 9.12 |  |
| E_max_ on ExGA, $\theta$_ExGAEMAX_ (%) | -100 | Fixed |  |
| E_max_ on HAQ, $\theta$_HAQEMAX_ (%) | -71.2 | 11.9 |  |
| E_max_ on MMT8, $\theta$_MMT8EMAX_ (%) | 18.5 | 21.5 |  |
| E_max_ on TIS PhGA, $\theta$_TISPhGAEMAX_ (%) | -100 | Fixed |  |
| *Additive Residual Unexplained Variability* |  |  |  |
| Additive RUV for CDASI-D, $\theta$_CDASIDRUV_ | 1.43 | 3.24 | 6.3 |
| Additive RUV for CDASI-A, $\theta$_CDASIARUV_ | 4.68 | 3.4 | 8.37 |
| Additive RUV for SF-36 PFD, $\theta$_SF36PFDRUV_ | 4.68 | 4.31 | 11.9 |
| Additive RUV for SF-36 MCS, $\theta$_SF36MCSRUV_ | 5.76 | 4.33 | 10.9 |
| Additive RUV for PhGA, $\theta$_PhGARUV_ | 12 | 3.26 | 5.77 |
| Additive RUV for PtGA, $\theta$_PtGARUV_ | 15.4 | 4.93 | 5.86 |
| Additive RUV for Enzymes, $\theta$_EnzymeRUV_ | 0.352 | 3.52 | 6.82 |
| Additive RUV for ExGA, $\theta$_ExGARUV_ | 1.2 | 8.02 | 6.47 |
| Additive RUV for HAQ, $\theta$_HAQRUV_ | 19 | 5.23 | 2.81 |
| Additive RUV for MMT8, $\theta$_MMT8RUV_ | 7.32 | 10.2 | 12 |
| Additive RUV for TIS PhGA, $\theta$_TISPhGARUV_ | 1.04 | 8.58 | 4.93 |
| *Variance-Covariance* |  |  |  |
| IIV on E_max,norm_, $\omega^{2}$_IIVEMAXNORM_ | 1.7 (45%) | 25.2 | 20.9 |
| IIV on E_pla_, $\omega^{2}$_IIVPBO_ | 0.00934 (10%) | 25.5 | 16.8 |
| IIV on baseline CDASI-D, $\omega^{2}$_IIVCDASID_ | 0.58 (106%) | 18.6 | 4.93 |
| IIV on baseline CDASI-A, $\omega^{2}$_IIVCDASIA_ | 0.25 (55%) | 18.8 | 3.5 |
| IIV on baseline SF-36 PFD, $\omega^{2}$_IIVSF36PFD_ | 0.0735 (28%) | 17.6 | 1.96 |
| IIV on baseline SF-36 MCS, $\omega^{2}$_IIVSF36MCS_ | 0.0316 (18%) | 19.2 | 5.06 |
| IIV on baseline PhGA, $\omega^{2}$_IIVPhGA_ | 0.0901 (31%/37%) | 18.9 | 6.46 |
| IIV on baseline PtGA, $\omega^{2}$_IIVPtGA_ | 0.114 (35%) | 29.4 | 3.84 |
| IIV on baseline Enzymes, $\omega^{2}$_IIVEnzyme_ | 0.0583 (44%) | 17.9 | 5.29 |
| IIV on baseline HAQ/MMT8, $\omega^{2}$_IIVHAQMMT8_ | 0.235 (53%/52%) | 31.5 | 1.74 |
| IIV on baseline ExGA, $\omega^{2}$_IIVExGA_ | 0.0628 (33%) | 55.7 | 19.9 |
| MMT8 $\omega^{2}$ from HAQ, $\theta$_PROHAQMMT_ | -0.389 | 14.2 |  |
| Part ExGA $\omega^{2}$ from PhGA, $\theta$_PROExGAPhGA_ | 1 | Fixed |  |
| CDASI-A ~ CDASI-D, $\omega_{cov}$_RCDASID-CDASIA_ | 0.195 | 27.9 |  |
| SF-36 PFD ~ CDASI-D, $\omega_{cov}$_RCDASID-SF36PFD_ | -0.0227 | 111 |  |
| SF-36 PFD ~ CDASI-A, $\omega_{cov}$_RCDASIA-SF36PFD_ | 0.0465 | 38 |  |
| SF-36 MCS ~ CDASI-D, $\omega_{cov}$_RCDASID-SF36MCS_ | -0.00449 | 384 |  |
| SF-36 MCS ~ CDASI-A, $\omega_{cov}$_RCDASIA-SF36MCS_ | 0.0301 | 40.4 |  |
| SF-36 MCS ~ SF-36 PFD, $\omega_{cov}$_RSF36PFD-SF36MCS_ | 0.0213 | 31.3 |  |
| PhGA ~ CDASI-D, $\omega_{cov}$_RCDASID-PhGA_ | 0.0454 | 66.1 |  |
| PhGA ~ CDASI-A, $\omega_{cov}$_RCDASIA-PhGA_ | 0.0795 | 27.3 |  |
| PhGA ~ SF-36 PFD, $\omega_{cov}$_RSF36PFD-PhGA_ | -0.0063 | 164 |  |
| PhGA ~ SF-36 MCS, $\omega_{cov}$_RSF36MCS-PhGA_ | 0.00251 | 279 |  |
| PtGA ~ CDASI-D, $\omega_{cov}$_RCDASID-PtGA_ | 0.00675 | 634 |  |
| PtGA ~ CDASI-A, $\omega_{cov}$_RCDASIA-PtGA_ | 0.0221 | 121 |  |
| PtGA ~ SF-36 PFD, $\omega_{cov}$_RSF36PFD-PtGA_ | -0.0159 | 95.2 |  |
| PtGA ~ SF-36 MCS, $\omega_{cov}$_RSF36MCS-PtGA_ | -0.00733 | 133 |  |
| PtGA ~ PhGA, $\omega_{cov}$_RPhGA-PtGA_ | 0.0448 | 42.4 |  |
| Enzymes ~ CDASI-D, $\omega_{cov}$_RCDASID-Enzyme_ | -0.000605 | 3850 |  |
| Enzymes ~ CDASI-A, $\omega_{cov}$_RCDASIA-Enzyme_ | -0.00471 | 330 |  |
| Enzymes ~ SF-36 PFD, $\omega_{cov}$_RSF36PFD-Enzyme_ | -0.00911 | 91.2 |  |
| Enzymes ~ SF-36 MCS, $\omega_{cov}$_RSF36MCS-Enzyme_ | 0.0104 | 54.9 |  |
| Enzymes ~ PhGA, $\omega_{cov}$_RPhGA-Enzyme_ | 0.00218 | 436 |  |
| Enzymes ~ PtGA, $\omega_{cov}$_RPtGA-Enzyme_ | -0.00836 | 158 |  |
| HAQ/MMT8 ~ CDASI-D, $\omega_{cov}$_RCDASID-HAQMMT8_ | -0.0113 | 521 |  |
| HAQ/MMT8 ~ CDASI-A, $\omega_{cov}$_RCDASIA-HAQMMT8_ | -0.0155 | 240 |  |
| HAQ/MMT8 ~ SF-36 PFD, $\omega_{cov}$_RSF36PFD-HAQMMT8_ | -0.0704 | 31.8 |  |
| HAQ/MMT8 ~ SF-36 MCS, $\omega_{cov}$_RSF36MCS-HAQMMT8_ | -0.0139 | 98.2 |  |
| HAQ/MMT8 ~ PhGA, $\omega_{cov}$_RPhGA-HAQMMT8_ | 0.0514 | 49.9 |  |
| HAQ/MMT8 ~ PtGA, $\omega_{cov}$_RPtGA-HAQMMT8_ | 0.0811 | 44.8 |  |
| HAQ/MMT8 ~ Enzymes, $\omega_{cov}$_REnzyme-HAQMMT8_ | 0.00807 | 214 |  |

For interindividual variability (IIV) estimates, the variance estimate is shown, with %CV of the resulting structural parameter in parentheses; when multiple structural parameters are described with a single parameter, the %CV for each are shown in order of displayed fixed effect, separated by slashes (for example, PhGA IIV %CV is shown as “PhGA %CV/TIS PhGA %CV”). Endpoint abbreviations are defined in Outcomes Assessments. RUV was modeled with fixed effect estimates for the standard deviations, so those estimates are shown before the random effect parameter estimates; the associated random effect parameters (sigmas) were all fixed to 1, so only the shrinkage parameter (based on standard deviation of weighted residuals) is shown with the corresponding RUV estimate.

Other Abbreviations: CI = confidence interval; CV = coefficient of variation; RSE = relative standard error; RUV = residual unexplained variability; SHR = shrinkage; SIR = sampling importance resampling.

### Supplementary Table S2. Naive initial model maximum effect parameter estimates.

| Endpoint | Estimate | Bootstrap | |
| --- | --- | --- | --- |
|  |  | Mean | RSE |
| CDASI-D | -18.5% | -14.6% | 164.6% |
| CDASI-A | -86.1% | -89.9% | 9.0% |
| SF-36 PFD | 12.9% | 14.8% | 91.3% |
| SF-36 MCS | 7.7% | 9.7% | 90.5% |
| PhGA | -92.6% | -94.9% | 5.3% |
| PtGA | -78.4% | -82.2% | 13.7% |
| Enzymes | -48.7% | -45.7% | 115.9% |
| ExGA | -99.5% | -95.4% | 9.5% |
| HAQ | -66.8% | -67.6% | 47.7% |
| MMT8 | 27.9% | 32.0% | 49.1% |
| TIS PtGA | -78.2% | -80.4% | 18.0% |
| TIS PhGA | -90.1% | -88.4% | 19.5% |

Estimates for the initial model without optimization for stability. No placebo effect was included in this model, and Total Improvement Score (TIS) Patient Global Assessment (PtGA) was treated as distinct from earlier PtGA (like Physician Global Assessment, PhGA), although they were the same. Bootstrap was performed to give covariance estimates despite model instability. Endpoint abbreviations are defined in Outcomes Assessments.

Other Abbreviations: RSE = relative standard error.

###

### Supplementary Table S3. Final model covariance parameter estimates.

| Parameter | CDASI-D | CDASI-A | SF-36 PFD | SF-36 MCS | PhGA | PtGA | Enzymes | HAQ/MMT8 |
| --- | --- | --- | --- | --- | --- | --- | --- | --- |
| CDASI-D | NA | 29.6 | 54.2 | 620 | 69 | 217 | 21300 | 722 |
| CDASI-A | 0.139 | NA | 168 | 34.8 | 24.2 | 612 | 96.9 | 205 |
| SF-36 PFD | -0.0435 | 0.00707 | NA | 29.7 | 101 | 50.8 | 310 | 34.7 |
| SF-36 MCS | -0.00272 | 0.0277 | 0.0203 | NA | 281 | 190 | 56.1 | 87.5 |
| PhGA | 0.0428 | 0.0724 | -0.00928 | 0.00247 | NA | 41.1 | 458 | 52.1 |
| PtGA | 0.0191 | 0.00333 | -0.0282 | -0.0049 | 0.045 | NA | 157 | 44.4 |
| Enzymes | -0.000109 | 0.013 | -0.00246 | 0.0101 | 0.00209 | -0.00815 | NA | 301 |
| HAQ/MMT8 | -0.00845 | 0.0142 | -0.0604 | -0.0159 | 0.0505 | 0.0801 | 0.00586 | NA |

Fitted covariance parameters between the interindividual variability estimates in the final model (Table 2). Estimates are shown in the lower triangle, with RSE percent in the upper triangle. Diagonal values are set to NA to avoid confusion. Endpoint abbreviations are defined in Outcomes Assessments.

###

### Supplementary Figure S1. Visual predictive checks for TIS Subscores.


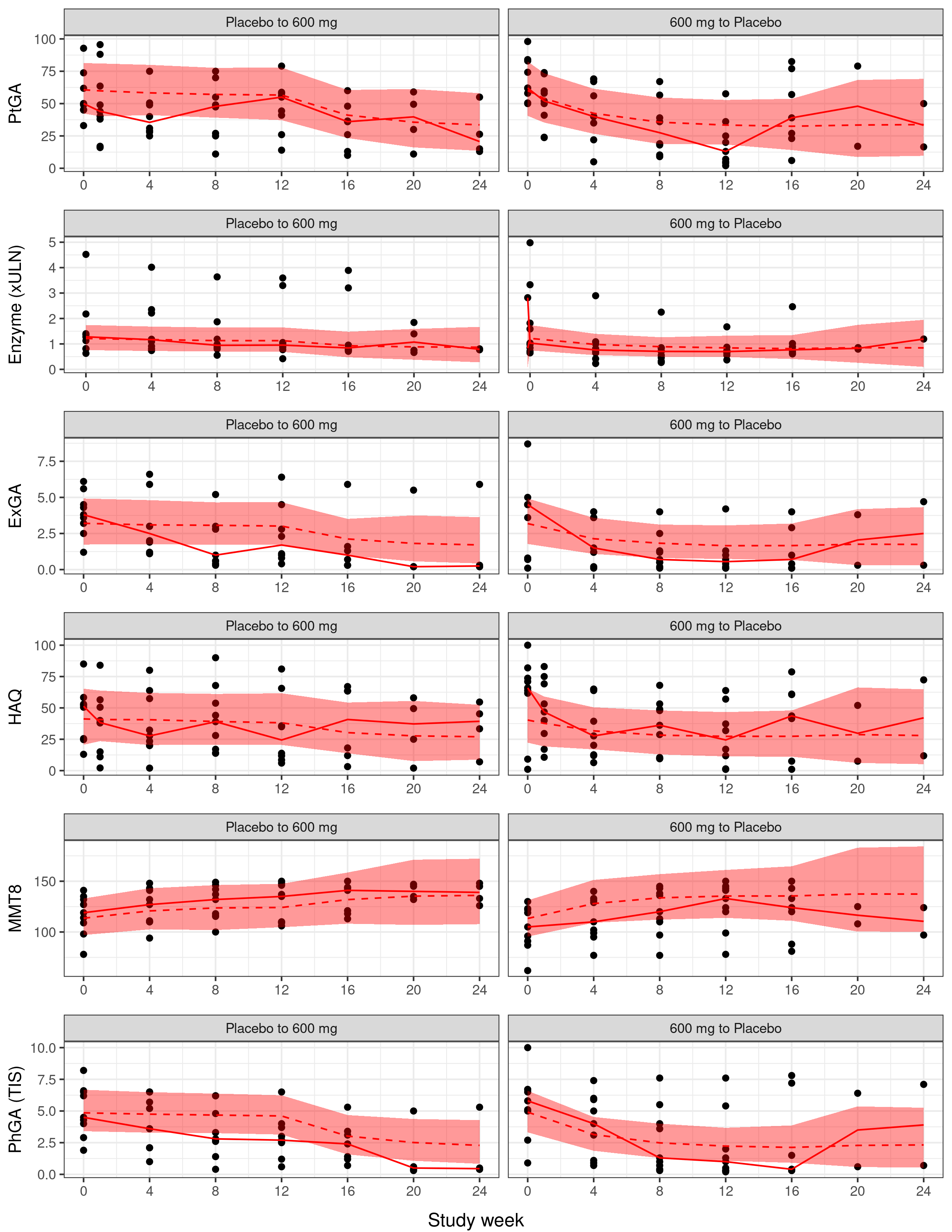


The plots in are continuous TIS subscores through end-of-study, with observed individual scores in black and median in red; medians and 95% confidence intervals from 1000 simulated trials are shown as dashed lines and bands. Only median is used for the predictive checks because of the small sample size. Endpoint abbreviations are defined in Outcomes Assessments.

### Supplementary Figure S2. Visual predictive checks for other endpoints.


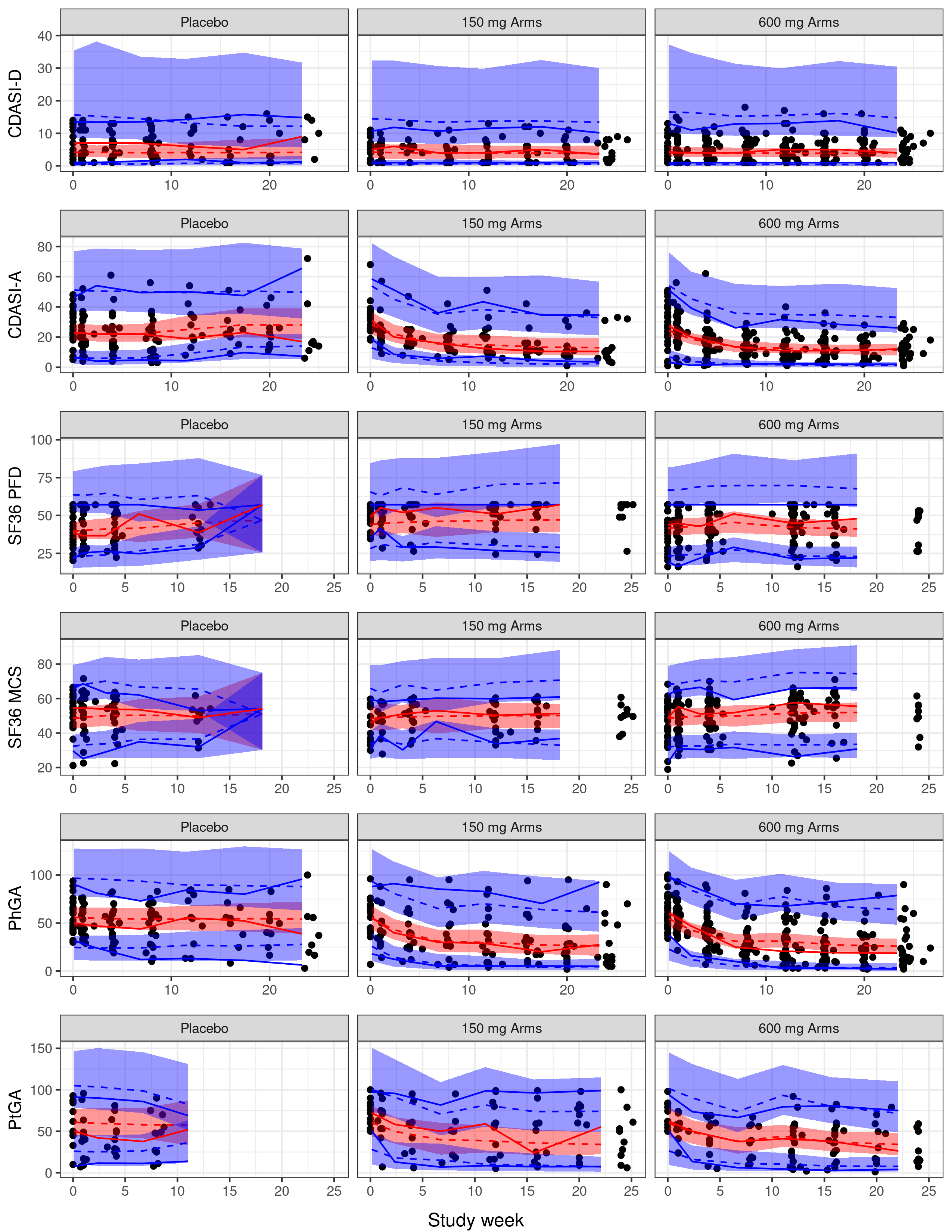


The plots in are continuous endpoint individual measurements in black, 95% distribution in blue, and median in red; 95% confidence intervals and medians for these quantiles from 1000 simulated trials are shown as bands and dashed lines. Points that seem to have no prediction bands are included but are before or after the center of the bin (where bands are drawn), with no subsequent connection point. Endpoint abbreviations are defined in Outcomes Assessments.

### Supplementary Figure S3. *Posthoc* predictions for baseline Manual Muscle Testing.


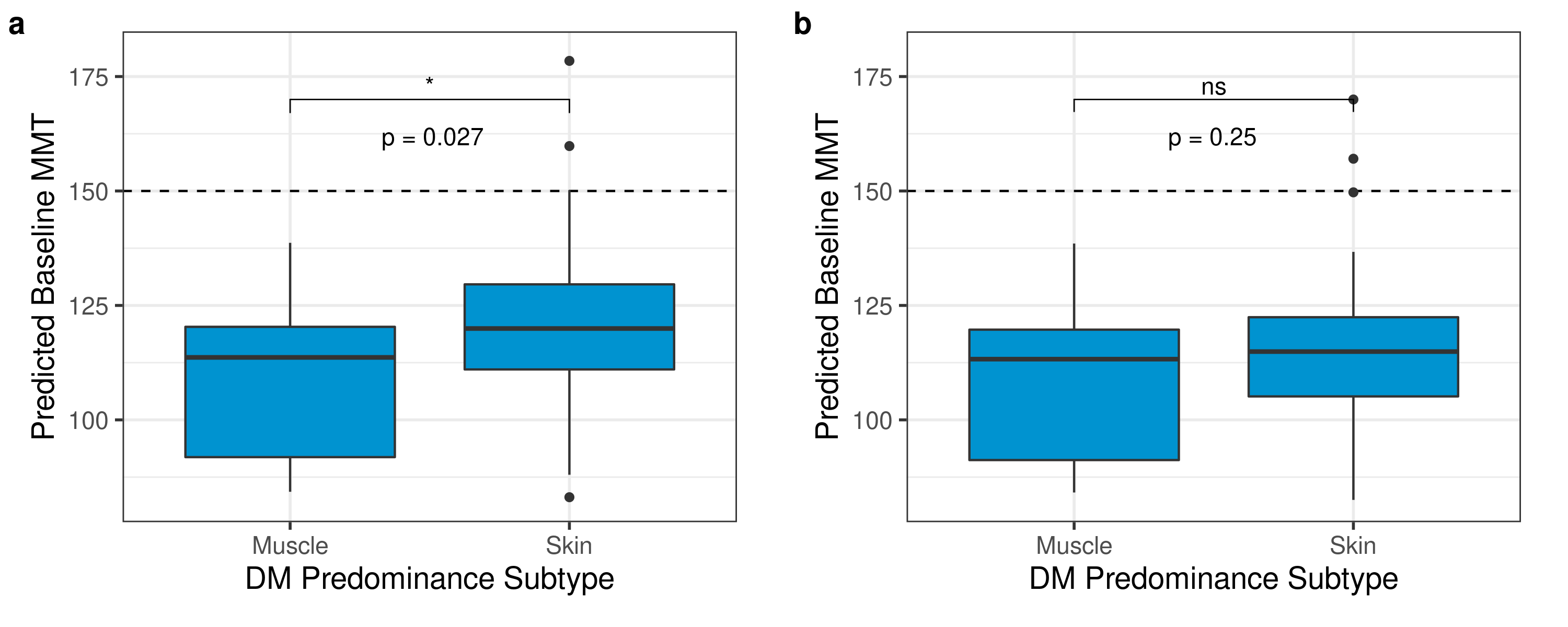


Comparison between skin- and muscle-predominant dermatomyositis (DM) *posthoc* estimates of baseline MMT-8 values from the base model (a) and final model (b). Significance is tested with an unpaired t-test to demonstrate differences; “*” denotes p < 0.05 and “ns” indicates not significant ($\alpha$ of 0.05 was not prespecified). Maximum MMT-8 is marked with a dashed line; there were no controls to account for the bounded nature of MMT-8. Endpoint abbreviations are defined in Outcomes Assessments.
